# Mr. Mackenzie on Strabismus
*On the Diseases of the Eye, p. 249, et seq.


**Published:** 1831

**Authors:** 


### XLIX.

### Mr. Mackenzie on Strabismus.*

Strabismus is an affection respecting which practitioners will frequently be consulted by anxious parents, and it possibly may redound to their advantage if they are acquainted with the current information on the subject. It is on this account that we are induced to notice Mr. Mackenzie's section upon it.

We need scarcely inform our readers that in strabismus one eye only is usually unsound. This is commonly directed inwards, sometimes outwards; the former is termed strabismus convergens, the latter strabismus divergens. In some the eyes squint alternately, or even both together. The vision of a squinting eye is almost always imperfect; those who squint with both eyes see confusedly; those who squint inwards with both are generally very short sighted.

The remote causes of strabismus are numerous. 1. It appears to arise in many instances from improper education of the eyes in young children, as from nurses laying the child in such a position in its cradle that it sees the light or any other remarkable object with one eye only, and so forth. Strabismus divergens is attributed to the improper practice of accustoming a child to look at the same time at two objects of which it is fond, at the light, for instance, on one side and the nurse on the other. 2. Children occasionally become squinters from the habit of looking at the point of their nose. 3. Imitation is a cause of squinting. 4. Covering a weak eye, which had been accidentally diseased, before the habit of observing objects with both eyes is perfectly established. 5. Spasm of the rectus, which may arise from a variety of causes. Mr. M. saw a little boy who was affected with strabismus immediately after squirting the oily juice of orange-skin into his eye, which produced great pain. 6. A speck of the cornea, by the habit which it occasions of the patient's turning the eye out of the natural axis of vision, in order that he may see past the speck. 7. The most frequent cause is imperfect vision from short sightedness or congenital defect in the retina. The distorted eye is in almost every case *very considerably*

* On the Diseases of the Eye, p. 249, et seq.

inferior in power to the other. 8. Strabismus is induced by various diseases of the brain, cerebral irritation from worms, teething, &c. Amaurosis affecting both eyes is generally attended with slight strabismus. 9. Whatever be the remote cause of strabismus, we cannot doubt that its proximate cause must in some way or other affect the muscles of the eyeball. One or more of these muscles must be in a state rendering them incapable of their natural exercise. The muscular substance may be in a state of atony, or the nervous energy which ought to animate them, may be imperfectly supplied. In by far the greater number of cases of strabismus, the eye rolls involuntarily inwards, which may lead us to conclude, that the abductor is in a state of unfitness for its office. It is not absolutely paralyzed, for on closing the sound eye it evidently exerts its proper function, but from some cause to us unknown, as soon as the sound eye is again opened, the muscular force of the abductor is no longer able to support the eye in its natural direction, so that the distortion immediately returns.

*Treatment.* "1. Our first object in the treatment of strabismus, must be to discover the cause. When this is accomplished, the plan of cure will be obvious; or, perhaps, we shall find reason to consider the defect as irremediable.

2. As strabismus often arises in children from abdominal irritation, we ought first to try the effect of an active purge or two; and then follow this up by mild aperients, and a carefully regulated diet. Squinting children are generally weakly, and often strumous, so that a course of tonic medicine will probably be useful.

3. Strabismus is frequently observed in children to be connected with a careless employment of the eyes, which is instantly corrected by exciting their attention. In other cases, the squint is never observed except when the child is in bad temper.

4. When only one eye squints, and when the defect in the sight of that eye is not very great, much may be done by strengthening its muscles, to cure the strabismus. The strengthening of the muscles is effected chiefly by tying up the sound eye, and thus obliging the patient to exercise only the eye which squints. Whenever the sound eye is blindfolded, the weak eye recovers its natural position in the orbit, and its natural motions. The patient finds that the sight gradually improves by use; and we observe that though the strabismus does return, on again exposing the sound eye, yet it is not to the same extent, and day after day becomes less, if the plan of cure is continued.

The patient need not keep the sound eye covered during the whole day. At first, this may be done for half an hour or an hour at a time, and then for longer periods. During the blindfolding of the sound eye, the weak one is to be exercised both on distant and on near objects, but especially on the former. If the patient be a child, he must be encouraged to exercise the weak eye in playing at ball or shuttlecock, viewing extensive prospects in the country, reading books printed in a large type, looking at prints, &c. Many authorities might be produced in favour of the efficaciousness of this mode of cure. Beer tells us, that by binding up the sound eye every day even for a couple of hours only, he had, in most cases, been successful.* It is worthy of remark, however, that this plan of curing strabismus is often attended by a diminished power both of motion and of vision in the sound eye; and that it has sometimes happened, that the squinting eye being cured by perseverance in this method, the sound eye has then become distorted. If both eyes squint from the first, they must be blindfolded alternately, each for several days at a time.

Another method of exercising the weak eye is that recommended by Dr. Jurin, in his Essay on Distinct and Indistinct Vision. Having placed the patient before us, we bid him close the undistorted eye, and look at us with the other. When we find the axis of this

* "Pflege gesunder und geschwächter Augen. p. 41. Frankfurt, 1802."

eye fixed directly upon us, we bid him endeavour to keep it in that situation, and open his other eye. Immediately, the distorted eye turns away from us towards his nose, and the axis of the other is pointed at us. But with patience and repeated trials, he will, by degrees, be able to keep the distorted eye fixed upon us, at least for some little time after the other is opened. When we have brought him to continue the axes of both eyes fixed upon us, as we stand directly before him, it will be time to change his position, and to set him first a little to one side of us and then to the other, and so to practise the same thing. When, in all these situations, he can perfectly and readily turn the axes of both eyes towards us, the cure is effected. An adult may practise all this in a mirror, without any director, though not so easily as with one.

5. As there is an inequality in the sensations of the sound and of the weak eye, it has been suggested that we should endeavour to render them more on a par, and that this of itself would tend to correct the distortion. Buffon recommended, therefore, that the patient should wear a pair of spectacles with a plane glass opposite to the bad eye, and a convex glass opposite to the good eye. In this way, the vision of the good eye would be rendered less distinct, and consequently it would be less in a state to act independently of the other.* As the weak eye is often short-sighted, perhaps the same advantage might be derived from placing a plane glass before the good eye, and a concave glass before the distorted one."

6. The treatment of strabismus must be varied as the cause is more or less connected with the muscles of the eye-ball. A merely bad habit may be overcome by the first two means. But discrimination is required. A girl had a speck on the cornea, occasioning strabismus. The speck was removed, but the squint remained. By a careful system of exercise, with the sound eye covered, a cure was effected. In strabismus convergens of both eyes, a pair of blinders, projecting in front from the temples, are recommended to be tried during a part at least of every day, with a view of attracting the eyes outwards. When the blinders are laid aside a broad green shade should be worn.

"Darwin employed a different plan, and with considerable success, in a case which appears to have partaken of the nature of this strabismus, and which he has related in the Philosophical Transactions. The patient was a child, of five years of age, exceedingly tractable and sensible. He viewed every object which was presented to him with but one eye at a time. If the object was presented on his right side, he viewed it with his left eye, and *vice versa.* He turned the pupil of that eye, which was on the same side with the object, in such a direction that the image of the object might fall on that part of the bottom of the eye where the optic nerve enters it. When an object was held directly before him, he turned his head a little to one side, and observed it with but one eye, *viz.* with that most distant from the object, turning away the other in the manner above described; and when he became tired with observing it with that eye, he turned his head the contrary way, and observed it with the other eye alone, with equal facility; but never turned the axes of both eyes on it at the same time. He saw and named letters, with equal ease, and at equal distances, with the one eye as with the other. There was no perceptible difference in the diameters of the irises, nor in their contractility, after having covered his eyes from the light. From these circumstances, Darwin was led at first to conclude that there was no defect in either eye,* but that the

* "Dissertation sur la Cause du Strabisme. Mémoires de l'Academie des Sciences pour 1743, p. 338. 12mo, Amsterdam, 1748."

* "From a series of experiments which he afterwards made, he came to the conclusion that the insensible spot at the bottom of this child's eye was four times the area of that in the eyes of others."

disease was simply a depraved habit of moving his eyes, which might probably be occasioned by the form of a cap or head-dress, which might have been too prominent on the sides of his face, like bluffs used on coach-horses, and might, in early infancy, have made it more convenient for the child to view objects placed obliquely with the opposite eye, till by habit the adductores were become stronger, and more ready for motion than their antagonists. Darwin recommended a paper gnomon to be made, and fixed to a cap. When this artificial nose was placed over his real nose, so as to project an inch between his eyes, the child, rather than turn his head so far to look at oblique objects, immediately began to view them with that eye which was next to them. The plan of cure was not persisted in; so that, six years after, Darwin found all the circumstances of this child's mode of vision exactly as they had been, except that they seemed established by longer habit, so that he could not bend the axes of both his eyes, on the same object, not even for a moment. By Darwin's advice, a gnomon of thin brass was made to stand over his nose, with half a circle of the same metal to go round his temples. These were covered with black silk, and by means of a buckle behind his head, and a cross piece over the crown of his head, this gnomon was worn without inconvenience, and projected before his nose about two inches and a half. By the intervention of this instrument, he soon found it less inconvenient to view oblique objects with the eye next to them, instead of the eye opposite to them. After this habit was weakened by a week's use of the gnomon, two bits of wood, about the size of a goose-quill, blackened all but a quarter of an inch at their summits, were frequently presented for him to look at, one being held on one side the extremity of the gnomon, and the other on the other side of it. As he viewed these, they were gradually brought forwards beyond the gnomon, and then one was concealed behind the other. By this means, in another week, he could bend both his eyes on the same object for half a minute together. By the practice of this exercise, before a glass, almost every hour in the day, he became in another week able to read for a minute together, with his eyes both directed on the same objects. By perseverance in the use of the artificial nose, he acquired more and more the voluntary power of directing both eyes to the same object, particularly if the object was not more than four or five feet from him, so that Darwin anticipated a complete cure."

In strabismus divergens, affecting both eyes, the alternate blindfolding of the eyes is likely to be useful. It has been recommended to apply a piece of black plaster on the point of the nose. Weller recommends a short funnel, made of pasteboard, with an oval base, to be so applied as to include both eyes, and having, at the part which rests above the point of the nose, an opening about an inch in diameter. Through this, fixed straight and firm, the patient must look, and by-and-bye read, when he must necessarily turn the eyes inwards and downwards.

